# Alfred Grace, M.R.C.S., L.S.A.

**Published:** 1916-07

**Authors:** 


### ALFRED GRACE, M.R.C.S., L.S.A.

WITH very sincere regret we have to record the deaths of the last two sons of the late Dr. Henry Mills Grace, of Downend, which have taken place during the last year. All five of the sons entered the profession at the Bristol Medical School, and all came well to the front in one or other of the fields of sport, at least three of them establishing records that will last for many years to come. The remarkable energy that pervaded the whole family enabled them not only to attend to their professional duties, but to find time to indulge to the full and to excel in any of the manly sports that they individually took up.

Alfred Grace, of Chipping Sodbury, was the second son, and was born in May, 1840. After attaining man's estate he qualified M.R.C.S. and L.S.A. in 1863 and 1864, and then acted as House Surgeon at the Bristol Royal Infirmary for six months.

In 1865 he went to Chipping Sodbury, taking the practice of the late Dr. Brookman, and was appointed Medical Officer to the Union and Public Vaccinator, which post he held till he resigned at the age of 65. He succeeded his father as Surgeon to the Royal Gloucestershire Hussars, and rose to the rank of Lieut.-Colonel. He was Certifying Factory Surgeon for the district and Deputy Coroner for the Lower Division of Gloucestershire Medical Officer to the Coalpit Heath Collieries (this post, now held by Mr. A. H. Grace, has been held by one of the family for eighty years), and he was doctor to the Oddfellows, Foresters, and many other clubs.

Some years ago he wrote an article in the British Medical

Journal, advocating large doses of morphia hypodermically in cases of eclampsia, which drew forth considerable correspondence in the medical journals of the time, and he was a man of sound judgment and dexterity in the many surgical cases that fell to his lot.

With all these appointments, an extensive family practice (his books averaged for years one hundred and fifty confinements per annum), and a large county connection among friends that he met in the hunting-field, it is marvellous that he was able to get through all that he did.

He was a good boxer, and quite a good cricketer, though he never rose to the eminence that his brothers enjoyed in that game. But if he did not take as high a place in cricket, he was about as good a rider to hounds as his brother W. G. was a cricketer. Many historic runs he took part in, and he once on an Irish thoroughbred cleared a stream near Littleton-on-Severn thirty feet from bank to bank. For thirty years he never bought a hunter, these being given him by various hunting friends, sometimes as unmanageable, but more often by admirers of his prowess in the saddle. He is frequently referred to by Whyte Melville and other hunting authors, in one place as the hunting doctor, worthy brother of the great W. G.

He was a man of retentive memory, and with his large experience of life and people in every grade of society, his practical knowledge of sport, and his keen appreciation of everything that makes life worth living, he was a most interesting companion, and his loss is felt by a large circle of friends.

